# Supplementary material for: The Evolution and Characterization of the RNA Interference Pathways in Lophotrochozoa
Source: Genome Biol Evol. 2024 May 7;16(5):evae098. doi: 10.1093/gbe/evae098 (PMC11114477; doi:10.1093/gbe/evae098)
Supplement: evae098_Supplementary_Data [file evae098_supplementary_data.zip › Supplementary_materials.zip!/selected_miRNAs/CrGi_selected_miRNAs/CrGi_NC_047561.1_10374.pdf]

Provisional ID	:	NC_047561.1_10374
Score total	:	3174.1
Score for star read(s)	:	3.9
Score for read counts	:	3168.2
Score for mfe	:	1
Score for randfold	:	1.6
Score for cons. seed	:	-0.6
Total read count	:	6226
Mature read count	:	5983
Loop read count	:	0
Star read count	:	243

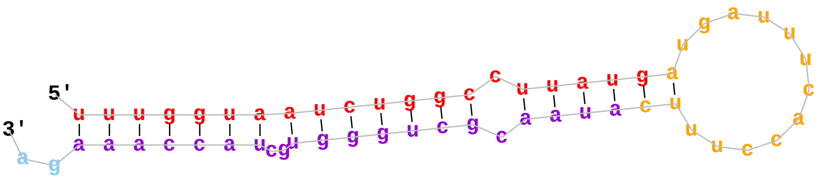

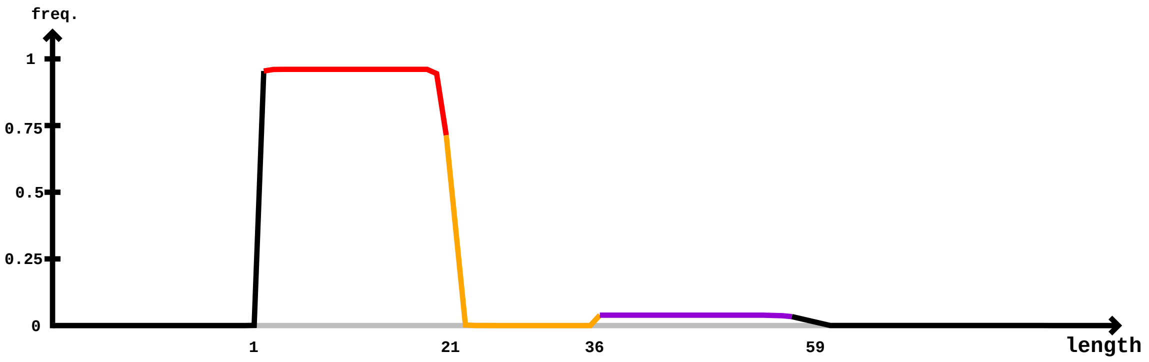


```
                                      Mature                              Star
5'- auauuugacugugcuuuuucuuugguaaucuggccuuaugaugauuucaccuuucauaacgcugggugcuaccaaagaccuucgcacggcacaagcgucaguuucagca  -3'   obs
    auauuugacugugcuuuuucuuugguaaucuggccuuaugaugauuucaccuuucauaacgcugggugcuaccaaagaccuucgcacggcacaagcgucaguuucagca        exp
    .....((.((((((....((((((((((((((((.((((((............)))))).)))))))..))))))))).....)))))))).((((....)))).....   reads    mm    sample
    ...................Uuugguaaucuggccuuau.......................................................................   1        1     Ov1
    ....................uuugguaaucuggccuua.......................................................................   29       0     Ov1
    ....................uuugguaaucuCgccuuau......................................................................   1        1     Ov1
    ....................uuugguaaucuggccuGau......................................................................   1        1     Ov1
    ....................uuugguaaucuggccuuaG......................................................................   1        1     Ov1
    ....................Cuugguaaucuggccuuau......................................................................   1        1     Ov1
    ....................uuugguaaucuggccuuau......................................................................   461      0     Ov1
    ....................uuugguaCucuggccuuaug.....................................................................   2        1     Ov1
    ....................uCugguaaucuggccuuaug.....................................................................   1        1     Ov1
    ....................uuugguaaucuggccuuUug.....................................................................   2        1     Ov1
    ....................uuugguaaucuggccuuaCg.....................................................................   1        1     Ov1
    ....................Guugguaaucuggccuuaug.....................................................................   1        1     Ov1
    ....................uuugguaaucCggccuuaug.....................................................................   1        1     Ov1
    ....................uuugguaaucuggccuuGug.....................................................................   1        1     Ov1
    ....................uuugAuaaucuggccuuaug.....................................................................   3        1     Ov1
    ....................uuugguaaucuggccuuaug.....................................................................   1361     0     Ov1
    ....................uuugguaaucuggcGuuaug.....................................................................   1        1     Ov1
    ....................uuugguaaCcuggccuuaug.....................................................................   1        1     Ov1
    ....................Cuugguaaucuggccuuaug.....................................................................   2        1     Ov1
    ....................uuugguaaucugAccuuaug.....................................................................   1        1     Ov1
    ....................uuugguaaucuggccuuauC.....................................................................   1        1     Ov1
    ....................uuCgguaaucuggccuuaug.....................................................................   1        1     Ov1
    ....................uuugCuaaucuggccuuaug.....................................................................   4        1     Ov1
    ....................uuugguaaucuggccuuauga....................................................................   47       0     Ov1
    ....................uuCgguaaucuggccuuauga....................................................................   1        1     Ov1
    ....................uuugguaaucuggccuuaugau...................................................................   2        0     Ov1
    ....................uuugguaaucuggccuuaugaA...................................................................   1        1     Ov1
    ....................uuugguaaucuggccuuaugauga.................................................................   1        0     Ov1
    .....................uugguaaucuggccuuau......................................................................   3        0     Ov1
    .....................uugguaaucuggccuuaug.....................................................................   7        0     Ov1
    ......................ugguaaucuggccuuaug.....................................................................   1        0     Ov1
    .......................................................auaacgcugggugcuacc....................................   4        0     Ov1
    .......................................................auaacgcugggugcuacca...................................   5        0     Ov1
```

```
                    Mature                            Star
auauuugacugugcuuuuucuuugguaaucuggccuuaugaugauuucaccuuucauaacgcugggugcuaccaaagaccuucgcacggcacaagcgucaguuucagca
.......................................................auaacgcugggugcuaccaa..................................    6     0    Ov1
.......................................................auaacgcugggugcuaccaaG.................................    1     1    Ov1
.......................................................auaacgcugggugcuaccaaa.................................    47    0    Ov1
.......................................................auaacgcugggugcuacUaaa.................................    1     1    Ov1
.......................................................auaacgcugggugcuaccaaag................................    31    0    Ov1
.......................................................auaacgcugggugcuaccaaaga...............................    2     0    Ov1
..............................................................................ccuucgcacggcacaagcgucagu.......    1     0    Ov1

....................uuugguaaucuggccuua.......................................................................    31    0    Ov2
....................Cuugguaaucuggccuuau......................................................................    1     1    Ov2
....................uuugguaaucuggccCuau......................................................................    1     1    Ov2
....................Auugguaaucuggccuuau......................................................................    1     1    Ov2
....................uuugguaaucuggccuuUu......................................................................    1     1    Ov2
....................uuugguaaucuggccuuau......................................................................    482   0    Ov2
....................uuugguaaucuggUcuuau......................................................................    1     1    Ov2
....................uuugAuaaucuggccuuaug.....................................................................    1     1    Ov2
....................uuugguGaucuggccuuaug.....................................................................    1     1    Ov2
....................uuugguaaucuggcUuuaug.....................................................................    1     1    Ov2
....................uuugguaaucuggccuuaug.....................................................................    1460  0    Ov2
....................uuugCuaaucuggccuuaug.....................................................................    2     1    Ov2
....................uuugguaauUuggccuuaug.....................................................................    1     1    Ov2
....................uuugguaaucuggAcuuaug.....................................................................    1     1    Ov2
....................uuugguaaucuggccuGaug.....................................................................    2     1    Ov2
....................uuuggCaaucuggccuuaug.....................................................................    1     1    Ov2
....................Guugguaaucuggccuuaug.....................................................................    2     1    Ov2
....................uuugguaaucCggccuuaug.....................................................................    2     1    Ov2
....................Auugguaaucuggccuuaug.....................................................................    2     1    Ov2
....................uCugguaaucuggccuuaug.....................................................................    1     1    Ov2
....................Cuugguaaucuggccuuaug.....................................................................    6     1    Ov2
....................uuugUuaaucuggccuuaug.....................................................................    2     1    Ov2
....................uuugguaaucuggccuuUug.....................................................................    2     1    Ov2
....................Cuugguaaucuggccuuauga....................................................................    1     1    Ov2
....................uuugguaaucuggccuuauAa....................................................................    1     1    Ov2
....................uuugguaaucuggccuuaugU....................................................................    1     1    Ov2
....................uuugguaaucuggccuuauga....................................................................    51    0    Ov2
....................uuugguaaucuggccuuaugaA...................................................................    2     1    Ov2
....................uuugguaaucuggccuuaugaG...................................................................    1     1    Ov2
....................uuugguaaucuggccuuaugau...................................................................    3     0    Ov2
.....................uuUguaaucuggccuuau......................................................................    1     1    Ov2
.....................uugguaaucuggccuuau......................................................................    5     0    Ov2
.....................uugguaaucuggccuuaug.....................................................................    6     0    Ov2
......................ugguaaucuggccuuaug.....................................................................    1     0    Ov2
.......................................................auaacgcugggugcuacc....................................    2     0    Ov2
.......................................................auaacgcugggugcuacca...................................    1     0    Ov2
.......................................................auaacgcugggugcuaccaU..................................    1     1    Ov2
.......................................................auaacgcugggugcuaccaa..................................    8     0    Ov2
.......................................................auaacgcugggugcuaccaaa.................................    48    0    Ov2
.......................................................auaacgcugggugcuaccaaag................................    35    0    Ov2
.......................................................auaacgcugggugcGaccaaag................................    1     1    Ov2

...................Uuuugguaaucuggccuuau......................................................................    1     1    Mt2
....................uuugguaaucuggccuua.......................................................................    13    0    Mt2
....................uuugguaaucuggccuuau......................................................................    238   0    Mt2
....................Guugguaaucuggccuuau......................................................................    1     1    Mt2
....................Cuugguaaucuggccuuau......................................................................    1     1    Mt2
....................uuugguaaucuggcUuuau......................................................................    1     1    Mt2
....................uuugguCaucuggccuuaug.....................................................................    1     1    Mt2
....................Cuugguaaucuggccuuaug.....................................................................    2     1    Mt2
....................uuGgguaaucuggccuuaug.....................................................................    1     1    Mt2
....................uuugguaGucuggccuuaug.....................................................................    1     1    Mt2
....................uuugguaaucuggccuuaCg.....................................................................    1     1    Mt2
....................uuugUuaaucuggccuuaug.....................................................................    1     1    Mt2
....................Guugguaaucuggccuuaug.....................................................................    3     1    Mt2
....................uuugguaaucuggGcuuaug.....................................................................    1     1    Mt2
....................uuugguGaucuggccuuaug.....................................................................    1     1    Mt2
....................uuugguaCucuggccuuaug.....................................................................    2     1    Mt2
....................uuugguaaucuggccuuUug.....................................................................    1     1    Mt2
....................uuugguaaucuggccuuaug.....................................................................    708   0    Mt2
....................uuugguaaucuggccuuauga....................................................................    28    0    Mt2
....................uuugguaaucuggccuuaugau...................................................................    2     0    Mt2
```

```
                    Mature                             Star
auauuugacugugcuuuuucuuugguaaucuggccuuaugaugauuucaccuuucauaacgcugggugcuaccaaagaccuucgcacggcacaagcgucaguuucagca
....................uuugguaaucuggccuuaugaA...................................................................   1     1     Mt2
.....................Gugguaaucuggccuuaug.....................................................................   1     1     Mt2
.....................uugguaaucuggccuuaug.....................................................................   9     0     Mt2
......................ugguaaucuggccuuaug.....................................................................   1     0     Mt2
.......................................................auaacgcugggugcuacc....................................   1     0     Mt2
.......................................................auaacgcugggugcuaccaa..................................   2     0     Mt2
.......................................................auaacgcugggugcuaccaaa.................................   11    0     Mt2
.......................................................auaacgcugggugcuaccaaag................................   8     0     Mt2

...................Uuuugguaaucuggccuuaug.....................................................................   2     1     Mt1
....................uuugguaaucuggccuua.......................................................................   27    0     Mt1
....................uuugguaaucuggccuuau......................................................................   235   0     Mt1
....................uuugguaaucuggccuuUug.....................................................................   1     1     Mt1
....................uuugguCaucuggccuuaug.....................................................................   1     1     Mt1
....................uuugUuaaucuggccuuaug.....................................................................   1     1     Mt1
....................Cuugguaaucuggccuuaug.....................................................................   1     1     Mt1
....................Guugguaaucuggccuuaug.....................................................................   2     1     Mt1
....................uuugguaaucuggccuuaug.....................................................................   640   0     Mt1
....................uuugguGaucuggccuuaug.....................................................................   2     1     Mt1
....................Auugguaaucuggccuuaug.....................................................................   2     1     Mt1
....................uCugguaaucuggccuuaug.....................................................................   1     1     Mt1
....................uuugguUaucuggccuuaug.....................................................................   1     1     Mt1
....................uuugguaaucuggccuuauga....................................................................   22    0     Mt1
....................uuugguaaucuggccuuaugau...................................................................   1     0     Mt1
.....................uugguaaucuggccuuaug.....................................................................   4     0     Mt1
.......................................................auaacgcugggugcuaccaa..................................   3     0     Mt1
.......................................................auaacgcugggugUuaccaa..................................   1     1     Mt1
.......................................................auaacgcugggugcuaccaaa.................................   18    0     Mt1
.......................................................auaacgcugggCgcuaccaaag................................   1     1     Mt1
.......................................................auaacgcugggugcuaccaaag................................   4     0     Mt1
.......................................................auaacgcugggugcuaccaaaga...............................   1     0     Mt1
```
